# Exploring barriers to the expanded programme on immunisation service utilisation in pastoralist communities of Afar, Afar Regional State, Ethiopia

**DOI:** 10.4102/hsag.v31i0.3230

**Published:** 2026-07-21

**Authors:** Mohammed A. Bilal, Robert T. Netangaheni

**Affiliations:** 1 Department of Health Sciences, College of Human Sciences, University of South Africa, Pretoria, South Africa

**Keywords:** barrier, Expanded Program on Immunisation, immunisation, vaccine, vaccination, pastoralist

## Abstract

**Background:**

Vaccination is a crucial public health intervention for reducing child mortality and morbidity, but Ethiopia and Africa have not met the Global Vaccine Action Plan’s target.

**Aim:**

The study aimed at exploring barriers to the expanded programme on immunisation service utilisation in pastoralist communities of Afar Regional State, Ethiopia.

**Setting:**

The research study was conducted in the Afar region of Ethiopia.

**Methods:**

A qualitative research approach was utilised, combining exploratory and descriptive designs with an appreciative inquiry method. The study focused on parents or guardians of children aged 12 months – 23 months who missed vaccines, along with Expanded Program on Immunisation (EPI) health workers, totalling 77 participants (60 parents and 17 healthcare providers). Data were collected through one-on-one interviews and focus group discussions, with the sample size determined by data saturation, employing non-probability purposive sampling.

**Results:**

Four main themes from the interview data include barriers to community immunization programs, such as geographical challenges, family mobility, vaccine hesitancy due to concerns about side effects, and general reluctance or ignorance regarding vaccination.

**Conclusion:**

The study identified barriers to community immunisation programmes, including geographical challenges, family mobility, vaccine hesitancy due to concerns about side effects and general reluctance or ignorance regarding vaccination.

**Contribution:**

This study provides context-specific insights into nursing care supervision, informing practice and policy for primary health care professionals in patient management and serving as a foundation for future research.

## Introduction

Immunisation is one of the vital strategies used to protect children from life-threatening diseases starting at birth. Therefore, vaccination is a weakened, living or deceased microorganism injected into a patient to activate their immune system against the microbe and ward off infectious diseases (Besnier et al. 2019). Vaccination is the most significant and economical public health measure in lowering child mortality and morbidity (Besnier et al. 2019). It is part and parcel of the process of immunisation, which the World Health Organization (WHO 2018) describes as a highly reliable and equally economical infectious disease prevention method. In Ethiopia, 43% of people are fully vaccinated (Melkamu et al. 2020a). Diseases that can be prevented by vaccination are serious public health issues that contribute significantly to child morbidity and mortality (Melkamu, Beyene & Zegeye 2020a).

A pastoralist community is often described as marginal, remote, conflict-prone, food insecure and associated with high levels of vulnerability (Zemariam et al. 2024). Pastoral communities of Ethiopia occupy 61% of the total land mass, and 97% of Ethiopian pastoralists are found in lowland areas of Afar, Somali, Oromia and Southern Nations, Nationalities and Peoples’ Region (SNNPR) (Zemariam et al. 2024).

According to a different global regular vaccination survey, 61% of children who were not vaccinated against the most common diseases during their first year of life reside in 10 countries, including Ethiopia (Nigatu et al. 2024). In addition, Africa has the highest percentage (17%) of children who are not immunised (Nigatu et al. 2024). Statistics showed that Africa and Ethiopia lagged behind the rest of the world and failed to achieve the global vaccine action plan (GVAP) 2011–2020 target. The GVAP 2011–2020 called for all countries to achieve 90% or more of the full national vaccination coverage by 2020 (WHO 2020). By 2015, the GVAP aimed to achieve at least 90% vaccination coverage worldwide and 80% Diphtheria-Pertussis-Tetanus 3 (DPT3) coverage in each district (WHO 2020).

In Ethiopia, diseases preventable by vaccinations are a major cause of morbidity and mortality among children under five (Moh.gov.et 2016). The major causes of child death in the country are meningitis (18%), pneumonia (18%), diarrhoea (18%) and measles (1%) (Moh.gov.et 2016). The Bacillus Calmette–Guérin (BCG), Measles, DPT-Hepatitis B-Hib (HepB + Hib) or Pentavalent, Rotavirus, Pneumococcus Vaccine (RPV) and Oral Polio Vaccine (OPV) are the 10 expanded programme on immunisation (EPI) vaccines that are currently offered in Ethiopia (Moh.gov.et 2016).

As per the Ethiopian Demographic and Health Survey (EDHS) report of 2019, the complete vaccination rate stands at 43% (Hailemeskel, Eshete & Shewasinad 2020). However, national studies between 2003 and August 2019 revealed that Ethiopia had a full vaccination rate of 58.92% (Hailemeskel et al. 2020). The immunisation rates throughout a large portion of the nation are below what is needed to create the necessary degree of herd immunity to stop the spread of eight illnesses targeted by the EPI (Hailemeskel et al. 2020). Similar regional differences exist in full vaccination coverage, which ranges from 12.6% in the Afar region to 75.8% in Addis Ababa (Hailemeskel et al. 2020).

Home delivery, residence, mother’s immunisation knowledge, health workers’ home visits, distance to medical facilities, skewed perceptions about the value of vaccinations and false beliefs about vaccine contraindications were all shown to be predictors of child immunisation (Nigatu et al. 2024). Additionally, studies have shown that vaccine coverage is influenced by the way health workers carry out their duties, the planning and execution of immunisation campaigns, the provision of services and the communication between parents and health professionals (Ketema et al. 2020). Nonetheless, insufficient or nonexistent immunisation of children is associated with systemic impediments (Ketema et al. 2020).

As mentioned by Olawuyi et al. (2023) in their studies, the government of Ethiopia has prioritised immunisation and, in recent years, has worked closely with its partners to increase the coverage in the Afar Region. It is important to understand the underlying causes of vaccine-preventable diseases as well as the reasons given by mothers and caregivers for choosing not to vaccinate their children; furthermore, this is especially true in nations where a significant proportion of children are not vaccinated (Olawuyi et al. 2023). Therefore, the researcher believes that examining the reasons behind immunisation discontinuation or lack thereof may aid in developing health system interventions aimed at enhancing immunisation coverage and, consequently, lowering vaccine-preventable illnesses in the Afar region of Ethiopia. The researcher is also mindful that: (1) people in this region strongly uphold traditional cultural values and beliefs and (2) live in highly traditional environments and recognise that some of these attitudes result in subpar EPI outcomes (Ketema et al. 2020). Thus, this study aimed to detect any barriers that may exist in the local context and to create plans for removing obstacles that stand in the way of the pastoralist communities in Ethiopia’s Afar Regional State, implementing EPI. Future researchers in this and/or related domains, health cadres, healthcare educators, policy makers and decision-makers are expected to use this work as a reference.

The study aimed to explore barriers to EPI service utilisation in pastoralist communities of Afar.

### Study setting

The study was conducted in the Afar Regional State which is located in North-Eastern Ethiopia. The Afar Regional State in Ethiopia is known for having the lowest full childhood immunisation coverage in the entire country. Similar regional differences exist in full vaccination coverage, which ranges from 12.6% in the Afar region to 75.8% in Addis Ababa (Eshete, Shewasinad & Hailemeskel 2020).

## Research methods and design

A mixed-methods approach, which integrates both qualitative and quantitative data collection, was most appropriate for this study as it allows for a more comprehensive understanding of the research problem (Dawadi, Shrestha & Giri 2021).

Using a qualitative approach, the researcher explored the barriers to implementing the EPI in pastoralist communities. Using both in-depth semi-structured interviews and focus groups in this qualitative study provided a more comprehensive understanding by leveraging their complementary strengths. In-depth interviews in this study allowed for deep, personal exploration of sensitive topics, while focus groups revealed shared experiences, group dynamics and social perspectives. It is worth noting that the Interviews offer detailed, individual insights, while focus groups are ideal for observing and discussing community-level interactions and perceptions. The study looked at both parents’ and healthcare providers’ perspectives to get a comprehensive understanding of the barriers to immunisation, as they face different challenges and have different views. Combining their perspectives provides a complete picture by identifying issues on both the ‘demand side’ (parental or community-level factors like misconceptions, distance or trust) and the ‘service-delivery or supply side’ (factors on the health system’s end, like staff shortages, vaccine availability or cold chain issues). This dual approach is crucial for designing effective and targeted strategies that address the needs of both the community and the health system.

### Population and sample

The population groups of interest were parents or guardians of children aged 12 months – 23 months who had missed one or more vaccines, such as the BCG vaccine, Pentavalent vaccine, Poliomyelitis vaccine, Measles vaccine, Pneumococcal Conjugate Vaccine, Rotavirus vaccine, and EPI health workers.

The sample size for this study was 77 participants, consisting of 60 parents or guardians and 17 healthcare providers, including health extension workers (HEWs) and partners working on EPI. Health extension workers are female, government-employed community health workers who form the backbone of Ethiopia’s Primary Health Care (PHC) system. They are the primary link between the formal health sector and households, especially in rural areas, and are widely credited for the country’s significant improvements in key health indicators over the past two decades. Health Extension Workers act as the primary link between the formal health system and the community, ensuring that immunisation services reach every eligible child. Their responsibilities span the entire process, from planning to follow-up. Their roles can be categorised into five key areas: community mobilisation, education, and demand creation; planning, registration, and defaulter tracing; logistics, cold chain management, and safety; vaccine administration and service delivery. Partners working in EPI are NGOs (non-governmental organisations) operating in various thematic areas to support government systems. In my research, these partners are NGOs specifically working within the EPI framework. Staff of those partners are involved in the interview. The HEWs are healthcare professionals stationed at health posts, employed by the government. Additionally, partners such as NGOs hire staff to support specific programs like EPI and enhance the system through logistics and technical assistance.

The interviewed participants’ sample size was determined by data saturation. The researcher used non-probability purposive sampling to choose participants.

The inclusion and exclusion criteria for participants involved in evaluating immunisation coverage among children aged 12 months – 23 months are described further. Inclusion criteria specify that eligible participants include parents or guardians of children within this age group who have missed one or more vaccines prior to the survey. In instances where vaccination cards were unavailable, maternal or caregiver recall was used to ascertain the child’s immunisation history. Furthermore, only those parents or guardians and children who are permanent residents of the study area and agree to participate will be included; residency is often indicated by the family’s prolonged presence within the community, particularly in pastoralist settings. Healthcare providers are also part of the inclusion criteria, notably those working in EPI services, including HEWs and partners who have provided health services in the study area for a minimum of 2 years. Conversely, the study outlines specific exclusion criteria as well. Parents or guardians of children who are already fully immunised will not be considered for the study, nor will healthcare providers who are not directly involved in EPI services, including HEWs and partners who do not deliver health services within the study area. Additionally, any potential participants who did not meet the stipulated inclusion criteria were excluded from the study.

### Data collection

In-depth semi-structured interviews and focus group discussions (FGD) yielded narratives that enabled the exploration of participants’ beliefs, attitudes and knowledge of strategies to overcome barriers to implementing the EPI. The study was piloted at the Ayseita district using an in-depth individual interview to assess and refine the data collection and analysis methods. Two healthcare workers and one parent participated in the pilot study. A pilot study allowed the researcher to familiarise themselves with the interview questions, facilitating free-flowing dialogue. It also allowed the researcher to practice using the digital voice recorder and taking field notes. The data obtained from the pilot study participants were not included in the main study. Data collection was conducted in two phases. Phase 1 was semi-structured interviews, and phase 2 was FGD interviews. Phase 1 allowed participants to speak openly about deeply personal or sensitive topics, such as cultural beliefs, personal fears or negative experiences with health services, which might not be openly shared in a group setting due to social desirability bias or fear of judgment. Phase 2, which is FGDs, leveraged group interaction to facilitate discussions about community-level barriers, collective beliefs and the social influences on health behaviours, such as community pressure regarding vaccination or shared perceptions of healthcare quality. Both phases of data collection were primary data collected by the researcher. Both the interview guides for individual in-depth semi-structured and FGDs were prepared by the researcher. The key questions for the individual interviews were key barriers in implementing the EPI in pastoralist communities, the geographic factors that impede the delivery of immunisation services, logistical and operational barriers also hinder EPI effectiveness, and various strategies implemented to address the challenges. Main discussions for the focus group interviews were challenges faced in accessing immunisation services for your child in pastoralist communities and cultural or social factors that influence your decision to vaccinate your child.

Permission to conduct the interview was requested from the head of the Woreda health office, which oversees all healthcare professionals, including HEWs, for approval. Request letters were written to the local area managers of the healthcare facilities. Time and dates for interviews were arranged, and the interviews were done from 05 October 2023 to 25 January 2024. In this study, interview questions were answered by study by the key participants and focus group participants, allowing them to describe their experiences in their own words. Translators conducted interviews in both Affaraf and Amharic, the native languages of the research area, and then translated the interviews back into English. To avoid misunderstandings and misinterpretations, the translators discussed the prepared English interview guide before heading out into the field. The focus groups are managed through careful preparation by the researcher and the assistant researcher. The researcher guided the discussions, using a guide with open-ended questions to meet research objectives while fostering a comfortable environment for participants to share their views. The assistant researcher managed logistics, recorded notes, and assisted the researcher during the sessions. Data were collected from 10 parents or guardians, 17 healthcare providers and five FGDs (each with 10 members).

Additional interview methods described by Polit and Beck (2021) included focus groups, semi-structured and in-depth interviews. A letter seeking permission to conduct the study was written to the head of the Woreda health office, which oversees all healthcare professionals, including HEWs. Attached to the permission letters was a copy of UNISA’s ethical clearance and authorisation ethical clearance and authorisation.

The researcher made sure that both the verbatim responses and the field notes complementarily helped with a re-enactment of the proceedings of each interview session. After the interview sessions, the researcher downloaded all digital copies and records onto a password-protected personal computer for safe and secure keeping. Data were collected from the Asayita, Afambo, Dubti, Semera logiya, and Mille districts.

### Data analysis

The study utilised an inductive thematic analysis to synthesise the recurring themes that emerged across the data.

The researcher transcribed data from the digital voice recorder and the field notes. The verbatim transcripts were sent to an independent co-coder to assist with data analysis. The co-coder was requested to sign a non-disclosure and service-level agreement with the researcher to ensure confidentiality. After the separate data analysis, the co-coder and the researcher discussed the coded data to reach consensus on the processes and possible conclusions.

The data collected from individual in-depth semi-structured interviews, FGDs, parents or guardians and healthcare providers were transcribed and organised into themes for presentation using an inductive thematic analysis of Braun and Clarke’s six phases of data analysis (Braun & Clarke 2021). The analysis of interviews and FGDs was merged by first applying Braun and Clarke’s six phases to each type of data separately, then systematically merging the codes and themes that emerged from both through a ‘focused coding’ stage, followed by a final review to ensure they were accurate and consistent with the research questions. This method guaranteed that while each data source provides distinct insights, a coherent, cohesive set of themes that reflect the whole dataset was generated.

### Trustworthiness

The standard of good qualitative research is based on trustworthiness. The researcher ensured trustworthiness by employing four constructs of trustworthiness, namely credibility, transferability, dependability and conformability (Tight 2019). To ensure credibility, the data were triangulated across the FGDs, semi-structured interviews and researcher field notes in order to find convergence. Data convergence was also attempted by using sample and eligibility criteria for participant selection in both the semi-structured interviews and the FGD. Transferability was ensured using a thorough description of the research setting and process. Dependability in this study was facilitated using a trail audit of all the processes and decisions undertaken. Audit trail means a detailed, transparent record of every step of the research process (Tight 2019). This means that the researcher systematically ensured and documented the consistency and trustworthiness of their work.

In addition, the reasons for the study processes were documented. The researcher also provided a detailed description of the research methods to ensure confirmability so that other researchers may duplicate the study.

### Ethical considerations

Ethical clearance was provided by the College of Health Studies Ethics Review Committee at UNISA, reference number: 14064316_CREC_CHS_2023. Subsequently, the researcher received approval from the Regional Health Bureau, which oversees district-level healthcare providers, including HEWs, and from the parents or guardians of the study participants’ children. All participants provided informed consent to participate in the study. The participation was voluntary, and the researcher also disclosed all pertinent aspects of the study to gain the trust and confidence of the participants. Before gathering data, the researcher made sure the participants understood the goal of the study. Before giving their agreement to participate in the study, the participants were fully informed about the risks, benefits and their rights. Every participant had the option to agree or reject taking part in the research project. Before starting a semi-structured interview, researchers obtained participants’ completed informed consent forms and explained the goal of the study.

## Results

This part presents the findings of the study, which aimed to identify obstacles to implementing the EPI in Afar pastoralist communities and explore potential solutions. Data were collected in October 2023 through in-depth interviews and FGDs, guided by a constructivist paradigm. Participants included parents, caregivers, health workers and focal persons, whose anonymised responses are presented below. The chapter begins with the biographical profiles of the 10 key informants.

The statements and responses of the interview participants were conceptually developed and evaluated, and are the main findings with their linked subthemes or categories discussed in this section. The study generated three main themes, as shown in Table 1.

**TABLE 1:** Summary of themes.

Theme	Subtheme
Theme 1: Participants’ general understanding of vaccine and vaccination	-
Theme 2: Community-side barriers for immunisation service provision in pastoralist setting	2.1: Accessibility barriers – Geographic location, transport and weather factors 2.2: Transportation problems are associated with geographic location 2.3: Migration of parents from one place to another 2.4: Fear of side effects 2.5: Parents’ and caregivers’ hesitancy
Theme 3: Healthcare system-side barriers for immunisation service provision in pastoralist setting	3.1: Lack of an adequate budget 3.2: Lack of transport for patients and healthcare providers 3.3: Lack of sufficient healthcare workforce 3.4: Lack of trained health workers

### Biographical characteristics of the participants

Table 2, Table 3 and Table 4 contain the biographical characteristics of the participants.

**TABLE 2:** Overall biographical data of participants for in-depth interviews (*N* = 10).

Pseudonym	Age (years)	Education	Gender	Language	District
Participant A	20	No	Female	Affaraf	Afambo
Participant B	45	No	Female	Affaraf	Afambo
Participant C	28	No	Female	Affaraf	Ayseita
Participant D	27	No	Female	Affaraf	Ayseita
Participant E	30	No	Female	Affaraf	Dubti
Participant F	28	No	Female	Affaraf	Dubti
Participant G	23	Grade 8	Female	Affaraf	Mille
Participant H	21	Grade 8	Female	Amharic	Mille
Participant I	18	Grade 7	Female	Affaraf	Semera logiya
Participant J	30	Grade 6	Female	Affaraf	Semera logiya

**TABLE 3:** Overall biographical data of participants for key informant interviews (*N* = 17).

Pseudonym	Age (years)	Gender	Experience (years)	Language	District
**Health extension workers**					
Participant K	22	Female	5	Affaraf	Afambo
Participant L	21	Female	4	Affaraf	Ayseita
Participant M	30	Female	4	Affaraf	Dubti
Participant N	19	Female	4	Amharic	Mille
Participant O	23	Female	3	Affaraf	Semera logiya
**Health centre EPI focal**					
Participant P	47	Male	14	Affaraf	Ayseita
Participant Q	25	Female	4	Affaraf	Dubti
Participant R	23	Female	1	Amharic	Mille
Participant S	32	Male	7	Amharic	Semera logiya
Participant T	23	Female	9	Affaraf	Afambo
**District focal**					
Participant U	29	Male	8	Amharic	Dubti
Participant V	36	Male	14	Amharic	Ayseita
Participant W	33	Male	7	Amharic	Afambo
Participant X	34	Male	13	Amharic	Mille
Participant Y	33	Male	10	Amharic	Semera logiya
Participant Z	32	Male	7	Affaraf	Regional-Semera
Participant AZ	45	Male	16	Amharic	Partner-Semera

EPI, expanded programme on immunisation.

**TABLE 4:** Overall biographical data of focus group discussions (*N* = 50).

Pseudonym	Language	District
FGD Z1	Affaraf	Afambo
FGD Z2	Affaraf	Ayseita
FGD Z3	Affaraf	Dubti
FGD Z4	Affaraf	Mille
FGD Z5	Affaraf	Semera logiya

FGD, focus group discussions.

### Theme 1: Participants’ general understanding of vaccines and vaccination

A lack of understanding directly hinders EPI utilisation. For instance, individuals who do not understand how vaccines work may fall prey to myths, such as ‘vaccines cause infertility’, leading to refusal or hesitation. This foundational knowledge is critical for identifying barriers to EPI service utilisation. By examining ‘participants’ general understanding of vaccines and vaccination’, it can be diagnosed the root cause, uncover the underlying factors affecting vaccine acceptance, provide essential context, understand the unique beliefs, myths and knowledge levels within the pastoralist communities of Afar.

Most participants from the in-depth interviews indicated that they had information about vaccines and vaccinations. However, some parents/guardian participants appeared to clearly lack understanding regarding what vaccines and vaccinations entail. The participants expressed mixed reactions: While some had negative experiences and attitudes towards vaccination and immunisation services, others had differing perspectives. The responses from the participants illustrate the range of experiences and views.

The 28-year-old mother (Participant C) stated that:

‘Vaccine is a medication that given for children’s up to 9 months. When we compare between children who received the vaccine and not received the vaccine, there is a difference. The vaccinated children are healthy, and they are … The unvaccinated ones, most of the time they become sick.’ (Participant C, 28, Female)

A 45-year-old mother (Participant B) stated that:

‘I know about vaccine; I think it is a service that is provided by health workers carrying vaccine carriers. Previously we were notified prior to the vaccination session, but nowadays we did not receive any information about the vaccination programme.’ (Participant B, 45, Female)

A common issue reported by the participants was a lack of information among mothers and caregivers. The KII participants all understood what vaccines and vaccinations are. However, newly hired or recently deployed staff were not adequately trained. The participants emphasised that it is crucial to educate these recently hired employees on the intervention areas of the programme. The responses from the participants are shown below:

A 23-year-old Health centre EPI focal with 1 year of experience (Participant R) stated that:

‘The newly hired staff members and those who have not received training are lacking in knowledge about the EPI. This lack of knowledge could potentially impact their ability to effectively carry out their responsibilities related to immunisation and public health.’ (Participant R, 23, Female)

The poor understanding of the importance and benefits of vaccination is a significant barrier to the successful implementation of vaccination programmes. This lack of understanding can lead to misconceptions, fear and reluctance among individuals and communities to participate in vaccination efforts. A 30-year-old HEW (Participant M), who had 4 years of expertise, stated that:

‘Our community assert that camel milk is an essential part of the answer to all this inside their communities. However, as I previously stated, when we inform them of the importance of the vaccine, they refuse and place the responsibility on us. Additionally, they have faith in traditional healers and frequently use the drugs prescribed by these healers – known locally as “Begi cora”. They try to massage the stomach area of their sick children since when they are ill, the movement of their intestines is the root of their trouble.’ (Participant M, 30, Female)

### Theme 2: Community-side barriers for immunisation service provision in a pastoralist setting

Evidence gathered from the in-depth interviews (IDIs), key informant interviews (KIIs) and FGDs revealed several barriers that contribute to children missing their full vaccination. The participants recounted and talked about these obstacles, which were grouped as subthemes.

#### Subtheme 2.1: Accessibility barriers – Geographic location, transport and weather factors

Some participants mentioned that these barriers include the geographical location of the residence, which may make it difficult for families to access vaccination services. The geographical location of a residence creates barriers to accessing vaccination services for families. This may be due to the limited availability of healthcare facilities in remote or rural areas, longer travel times to reach vaccination sites or a lack of transportation options. Additionally, families living in areas with limited healthcare infrastructure may face challenges accessing reliable information about vaccination schedules and availability.

The participants gave the following responses: A 36-year-old mother from the Z4 FGD group (Informant 1) stated:

‘I have personally neglected to vaccinate my children, a problem that most people in our community have because the health post is located far from the district.’ (Informant 1, 36, Female)

A 33-year-old mother from the Z4 FGD group (Informant 2) stated that:

‘There is also a remote, difficult-to-reach place, inaccessible to our community’s residents. Additionally, they haven’t received any information or cannot receive any service use.’ (Informant 2, 33, Female)

#### Subtheme 2.2: Transportation problems are associated with geographic location

The evidence from the participants’ responses also showed that factors such as floods and transportation problems can create additional barriers for families trying to reach vaccination centres. Most households responded that the distance between the village and the healthcare facility is their prime obstacle. This condition got worse during the floods when the road was damaged. The utilisation of healthcare services largely depended on access to transportation. In rural areas, when the distance to travel is long and no proper transportation facility is available, it ultimately leads to low utilisation of public healthcare services and an increase in out-of-pocket health expenditure. The following participants supported this:

A 29-year-old mother from the Z4 FGD group (Informant 5) stated that:

‘There is also a difficulty, a flood occurrence in our community. When a flood arises in our community, there will be the destruction of a road or result in transportation problems. Due to that majority of the community beyond the flood area will not obtain the vaccine session.’ (Informant 5, 29, Female)

A 27-year-old mother from the Z5 FGD group (Informant 8) stated that:

‘The health centre is far from our village. So, there is a payment fee for transportation. It is difficult to go to the health centre and back. It needs a fee to pay for the transportation that makes our vaccination of our child challenging.’ (Informant 8, 27, Female)

#### Subtheme 2.3: Migration of parents from one place to another

The participants mentioned that parents’ migration from one place to another and from one region to another was a barrier to full vaccination because some families live in the border area. When families move, it can disrupt the established vaccination schedule for their children, leading to missed or delayed doses. This can increase the risk of children being vulnerable to vaccine-preventable diseases. Additionally, relocating to a new area may also pose challenges in finding healthcare providers and navigating the new healthcare system, further impacting the timely vaccination of children. The participant gave the following response:

A 36-year-old district EPI focal who has 14 years of expertise (Participant V) reported that:

‘The majority of our communities are mobile, and many of them experience annual flooding. Thus, this results in the failure to receive or avoid vaccinations.’ (Participant V, 36, Male)

A 36-year-old district EPI focal who had 14 years of expertise (Participant V) said that:

‘Implementing an immunisation programme is primarily challenging due to the mobile lifestyle of the local population and a lack of financing or inadequate allocation for the district’s operations.’ (Participant V, 36, Male)

#### Subtheme 2.4: Fear of side effects

Participants identified barriers to completing the full vaccination schedule or missing the vaccination schedule for children, including fear of potential side effects. This fear often stems from misinformation or misconceptions about the safety of vaccines. Some parents may worry that vaccines could cause serious side effects or long-term health problems for their children. Concerns about pain or discomfort during the vaccination process can also contribute to hesitancy in completing the full schedule. The participants provided the following opinions:

A 22-year-old mother from the Z1 FGD group (Informant 4) reported that:

‘Additionally, some community members choose not to vaccinate or not to vaccinate their children because of possible adverse reactions to the vaccine, such as swelling or redness near the injection site.’ (Informant 4, 22, Female)

A 30-year-old mother (Participant E) stated that:

‘I vaccinated my young child in the hospital, but my child got sick after the shot, so I had to default or stop the shot out of concern for further risks.’ (Participant E, 30, Female)

#### Subtheme 2.5: Parents and caregivers’ hesitancy

Reluctance to get vaccinated or refusal to vaccinate one’s children is known as vaccine hesitation. The participants reported that vaccine hesitation in pastoralist groups may be caused by misconceptions about the safety and effectiveness of vaccines and cultural beliefs. A 30-year-old HEW (Participant M), who had 4 years of expertise, stated that:

‘Immunisation reluctance has been observed in our district, particularly in those who argue that the vaccine is only important for the immunisation provider’s survival.’ (Participant M, 30, Female)

### Theme 3: Healthcare system-side barriers for immunisation service provision in a pastoralist setting

The evidence gathered from the data collection revealed several barriers in public health facilities (child vaccination service delivery) that contribute to children missing or defaulting on full vaccination. Concerning the child vaccination service delivery thematic area, the evidence from the healthcare worker participants indicated that lack of budget, lack of vehicles, lack of sufficient health workforce and absence of HEWs in health posts were the reasons for missing vaccination.

#### Subtheme 3.1: Lack of an adequate budget

The evidence from the participants indicated that a lack of budget was a significant issue. This suggests that financial constraints were a major factor affecting the situation. The respondents’ feedback highlighted the impact of limited resources on their ability to address the issue at hand.

A 33-year-old district EPI focal who has 10 years of expertise (Participant Y) said that:

‘Additionally, there is a shortage of funding for outreach programmes as well as an absence of vehicle and motorbike services.’ (Participant Y, 33, Male)

#### Subtheme 3.2: Lack of transport for patients and healthcare providers

The evidence from the participants indicates that the lack of vehicles has been a significant challenge for them. The lack of vehicles has also limited their mobility and independence, making participating in various activities and opportunities difficult.

A 34-year-old district EPI focal person who had 13 years of expertise (Participant X) said that:

‘As to our catchment location, we have a logistic problem, and it is hard to reach an area, which is difficult to go by on foot to provide vaccines for children and due to lack of motorcycle for supervision to ensure that all the children were vaccinated. There is also a lack of refresher training on child vaccination for some health extension workers, and I believe these problems could be the barriers to child vaccination.’ (Participant X, 34, Male)

#### Subtheme 3.3: Lack of a sufficient health workforce

The evidence from the participants suggests that a lack of a sufficient health workforce is a significant barrier to vaccination programmes. This could mean insufficiently trained healthcare professionals are available to administer vaccines, educate the public about the importance of vaccination and provide follow-up care. This shortage of health workforce may hinder the effectiveness and reach of vaccination programmes, particularly in areas with limited access to healthcare services.

Another 25-year-old health centre EPI focal person (Participant Q) said that:

‘Of our catchment area, there is a shortage of human resources, especially health extension workers. At some health posts, only one health extension worker works with a large population and densely populated kebele. Therefore, only one health extension worker per kebele cannot reach every child in such difficult conditions. This is a major cause of child vaccine defaulting and not full vaccination.’ (Participant Q, 25, Female)

The information provided by the participants indicated that one of the barriers to the immunisation programme is the lack of HEW present at the workstation. This issue could hinder the effectiveness and reach of the immunisation programme, as well as the overall health outcomes for the population.

The 27-year-old mother (Participant D) reported that:

‘As to my child defaulting from getting full vaccination, one day when I brought my child for vaccination at 9 months, the health extension worker told me the vaccine is not opened only for your child and she said this vial can only be opened for 10 children. Again, one day, when I returned to the health post, it was closed, and I didn’t see a health extension worker at the post, so I didn’t return for vaccination next time.’ (Participant D, 27, Female)

Other evidence from the 28-year-old (Participant F) mother indicated that:

‘My child did not complete all recommended vaccines because the health extension worker of our kebele was not available at the health post. Because she had gone to attend her education, as I heard from kebele leaders, that is why my child did not receive all vaccines.’ (Participant F, 28, Female)

#### Subtheme 3.4: Lack of trained health workers

From the participants’ views, building capacity is a fundamental component of the immunisation programme. Accurate knowledge transmission is ensured, and the quality of the immunisation programme is directly supported by improving professional knowledge and skills through training and competency assessments.

A 21-year-old HEW (Participant L) said that:

‘As of right now, I have not gotten any training on the immunisation programme, although working as a vaccine provider.’ (Participant L, 21, Female)

## Discussion

The study’s overall findings add to the existing literature on barriers to EPI service utilisation. It examines the general understanding of vaccines and vaccination among participants, highlighting the prevalent lack of information among caregivers and its significant impact on immunisation rates. The findings from this study are not entirely unique – many of the barriers listed (distance, fear of side effects, workforce shortages) are common across low- and middle-income countries (LMICs). In the Afar pastoralist context, barriers differ significantly from those in sedentary or accessible LMIC settings, influenced by the unique nature of mobility, which is integral to their way of life rather than merely a matter of distance.

One typical LMIC barrier is the distance to a static health facility. In many rural agricultural communities, the challenge is that the village is 10 km from the nearest clinic. The solution is often to build a closer health post or provide ambulances. Afar pastoralist difference: The entire community and the health post itself are mobile. The barrier isn’t just that a family lives far from a health post; it’s that the family moves with their herds in search of water and pasture, making any fixed-point service delivery model obsolete. As Participant V stated, ‘The majority of our communities are mobile. This results in the failure to receive or avoid vaccinations’. This is a logistical challenge of a different magnitude.

The interpretation of findings was discussed according to each theme presented above. Lack of information with the mother or caregiver was a frequently reported factor. The findings further corroborated this observation as most of the caregivers participating in the study were unaware of immunisation’s benefits. Similar observations were reported in previous studies that explored reasons for incomplete immunisation and stated that lack of knowledge was the main reason (Saleh & Saleh 2022; Sharma & Singh 2021; Anand et al. 2025).

The researcher believes it is important to understand the perspective of pastoralist communities regarding immunisation. This understanding can provide valuable insights into their knowledge, attitudes and practices related to immunisation. It can also help identify potential barriers and challenges to ensure widespread immunisation coverage within pastoralist communities. By understanding their views on immunisation, targeted and culturally sensitive interventions can be developed to improve immunisation uptake and ultimately contribute to better public health outcomes for these communities. In this study, one key cultural issue is the pastoralist way of life, which involves moving with herds to find water and pasture. This lifestyle clashes with fixed health post locations and scheduled vaccination days, as families may be kilometres away on vaccination day. The structure of the health system inherently prioritises sedentary populations, rendering pastoralist communities ‘hard to reach’ and frequently excluded. To develop effective and culturally sensitive interventions for the EPI in pastoral areas, it is essential to deeply understand their distinct lifestyle and worldview.

A major contributing factor to the low accessibility of immunisation services was distance. According to the study by Eshete et al. (2020), some hard-to-reach locations lack access to health institutions that can administer childhood vaccinations. These findings are consistent with these findings. Families whose home was at least an hour from the vaccination site were less likely to be fully vaccinated (56%) than families whose home was between 30 min and 59 min away (67%) (Eshete et al. 2020). Similar findings were also reported by Fite and Hailu (2019), who found that moms chose to stop the kid’s immunisation regimen due to difficulty commuting or transporting the child to the health facility. It is noteworthy, therefore, that the estimated distance to the health institution affects immunisation propensity.

The utilisation of healthcare services largely depended on access to transportation. In rural areas, when the distance to travel is long and no proper transportation facility is available, it ultimately leads to low utilisation of public healthcare services and an increase in out-of-pocket health expenditure. Floods can make roads impassable and transportation difficult, especially in rural areas with limited infrastructure. A study by Fite and Hailu (2019) confirmed these findings, indicating that parents’ lack of awareness about children’s immunisations, irrational concerns about vaccine safety and lack of access to transportation for community and healthcare providers are some of the barriers to immunisation.

Furthermore, the evidence revealed that parents’ migration from one place to another can create barriers to accessing healthcare services, including vaccinations for their children. When families move, it can disrupt the established vaccination schedule for their children, leading to missed or delayed doses. A similar conclusion was drawn by Akwataghibe et al. (2019), who showed that migration was also a barrier to child vaccine coverage. Additionally, research by Njeru (2019), which was backed by the literature, showed that migrant groups frequently relocate, which makes it challenging to track vaccination status and lowers adherence to vaccination regimens. The studies conducted in Sinana District by De Figueiredo et al. (2020), in Somalia by Mohamud et al. (2020) and in Afghanistan by Pollard and Bijker (2020) supported this finding (Mohamoud et al. 2024). This could be due to similar sociodemographic characteristics of the population that can lead to defaulting of the children from full vaccination.

The study showed that vaccination service usage is influenced by fear of adverse effects that may occur after vaccination administration. The study’s results align with those of a study conducted in India by Vaghela et al. (2021), which found that poor immunisation coverage was caused by concerns about side effects and suggested that methods for improving services be adopted.

A study conducted in Nigeria by Malas and Tolsá (2022) supports the finding that community rumours and fear were factors in vaccine reluctance. Similar findings were also reported by De Figueiredo et al. (2020). Vaccine reluctance and distrust have been seen worldwide across socioeconomic strata, with environmental factors differing within and between nations.

These results are supported by research conducted in Ethiopia by Tagbo, Okafor and Okolie (2020), who found that one of the factors contributing to children’s partial immunisation is mothers’ lack of knowledge about the value of immunisation and the fact that most of them never took their children back to the doctor for follow-up vaccination because they were afraid of the side effects.

Concerning the child vaccination service delivery thematic area, the evidence from the participants indicated that lack of budget, lack of vehicles, lack of sufficient health workforce, absence of HEWs in health posts during visits by parents or caregivers and absence of HEWs from health posts were the reasons for defaulting vaccination.

A Pakistani study by Fahmida et al. (2022) found that limited budget, infrastructural deficits, inconsistent policy, health workforce and insufficient training result in low vaccination uptake. These findings by Fahmida et al. (2022) support this current research’s findings. Additionally, countries like Pakistan continue to struggle with efficiently using and transferring the allotted funding for immunisation (Fahmida et al. 2022). The evidence from the respondents indicated that the lack of vehicles has been a significant challenge for them. This has impacted their ability to commute to work, run errands and access essential services. The lack of vehicles has also limited their mobility and independence, making participating in various activities and opportunities difficult. Some studies also show numerous complaints about the poor funding for outreach initiatives, including the lack of motorbikes (Fahmida et al. 2022).

The evidence from the respondents suggests that a lack of sufficient health workforce is a significant barrier to vaccination programmes. This could mean insufficiently trained healthcare professionals are available to administer vaccines, educate the public about the importance of vaccination and provide follow-up care. This shortage of health workforce may hinder the effectiveness and reach of vaccination programmes, particularly in areas with limited access to healthcare services. One of the challenges facing the immunisation programme is the scarcity of human resources (Malande et al. 2019).

Building capacity is a fundamental component of the immunisation programme. Accurate knowledge transmission is ensured, and the quality of the immunisation programme is directly supported by improving professional knowledge and skills through training and competency assessments. This study is supported by research which indicates that healthcare provider barriers to immunisation include a lack of knowledge about indications for and contraindications to immunisations and poorly trained health staff (Fite & Hailu 2019).

The information provided by the respondents indicated that one of the barriers to the immunisation programme is the lack of HEWs present at the workstation. Other investigations revealed similar observations. According to Ketema et al. (2020), in addition to the irregularity of the outreach and scheduled sessions caused by the absence of vaccinators at the health facilities, poor accessibility and use were also a result of a lack of transportation options.

The researcher’s belief underscores the significance of understanding the various barriers from the community that lead to children missing or defaulting on full vaccination. Identifying and comprehensively analysing these barriers makes it possible to develop targeted interventions and strategies to address the specific challenges that hinder children from receiving complete vaccination. This approach can ultimately improve vaccination rates and overall public health outcomes.

### Study limitations and recommendations

This study used a qualitative methodology, allowing for an in-depth analysis of the underlying causes of children’s vaccine barriers, overcoming the constraints of earlier quantitative studies. The study’s comprehensive data collection, which included parents, carers and various layers of health professionals, including HEWs at the health post level, as well as regional, district and health centre EPI focal sites, was a major strength. This method allowed for a detailed knowledge of their common perspectives on service delivery, personal experiences with the programme and the problems they faced when offering EPI services. Furthermore, a validation workshop was held to finalise and agree on the newly formed strategic plan.

Various limitations to the qualitative research on vaccination were discussed in the report’s discussion section. Comparing the results with those from other papers became more difficult. Because there were so few qualitative publications, particularly on childhood immunisation, the research had to rely on mixed studies. This constraint may have influenced the extent and complexity of the debate, as well as its comparison to other studies.

From the study findings, several recommendations have been made. It is important to consider the local context and demographic characteristics while implementing health improvement programmes. The Federal Minister of Health ought to ensure that plans for reaching kebeles in pastoralist environments are developed, which should involve hiring enough medical personnel to shorten wait times and setting up mobile vaccination clinics to target pastoralist populations in isolated and difficult-to-reach locations. Attention should also be paid to the problem of women migrating or moving. Furthermore, there must be satellite clinics near or in pastoralist settlements so parents and caregivers can easily receive vaccinations. Accurate information should be provided in local languages to dispel myths and concerns about vaccinations. Additionally, capacity building programmes for medical staff on effectively addressing and interacting with parents who express concerns about the potential side effects of vaccines are necessary. Conducting outreach initiatives regularly will improve immunisation accessibility in remote locations. Finally, utilising the Dagu system, community gatherings and local media are examples of avenues through which information can be communicated that are acceptable in the pastoralist culture.

## Conclusion

After thoroughly examining the interview data, three main themes and associated sub-themes were found. The report also listed several barriers to the implementation of community immunisation programmes. Among these impediments were community-side barriers that hinder access to immunisation services, such as geographical challenges, transportation issues, and the effects of migration, fear of side effects, reluctance to vaccinate and poor understanding of vaccination. It also explores healthcare system-related barriers, including insufficient resources and workforce limitations. The research contributed creative ways to overcome access obstacles and increase the use of immunisation services.
